# Apatinib Induces Ferroptosis of Glioma Cells through Modulation of the VEGFR2/Nrf2 Pathway

**DOI:** 10.1155/2022/9925919

**Published:** 2022-05-11

**Authors:** Liang Xia, Mingjie Gong, Yangfan Zou, Zeng Wang, Bin Wu, Shuyuan Zhang, Liwen Li, Kai Jin, Caixing Sun

**Affiliations:** ^1^Department of Neurosurgery, The Cancer Hospital of the University of Chinese Academy of Sciences (Zhejiang Cancer Hospital) Institute of Basic Medicine and Cancer (IBMC), Chinese Academy of Sciences, Hangzhou 310022, China; ^2^Key Laboratory of Head & Neck Cancer, Translational Research of Zhejiang Province, Hangzhou 310022, China; ^3^Department of Neurosurgery, Changshu No. 2 People's Hospital, The Affiliated Changshu Hospital of Xuzhou Medical University, Changshu 215500, China; ^4^Department of Pharmacy, The Cancer Hospital of the University of Chinese Academy of Sciences (Zhejiang Cancer Hospital), Institute of Basic Medicine and Cancer (IBMC), Chinese Academy of Sciences, Hangzhou 310022, China

## Abstract

**Background:**

Glioma is a common tumor that originated from the brain, and molecular targeted therapy is one of the important treatment modalities of glioma. Apatinib is a small-molecule tyrosine kinase inhibitor, which is widely used for the treatment of glioma. However, the underlying molecular mechanism has remained elusive. Recently, emerging evidence has proved the remarkable anticancer effects of ferroptosis. In this study, a new ferroptosis-related mechanism of apatinib inhibiting proliferation of glioma cells was investigated, which facilitated further study on inhibitory effects of apatinib on cancer cells.

**Methods:**

Human glioma U251 and U87 cell lines and normal astrocytes were treated with apatinib. Ferroptosis, cell cycle, apoptosis, and proliferation were determined. A nude mouse xenograft model was constructed, and tumor growth rate was detected. Tumor tissues were collected to estimate ferroptosis levels and to identify the relevant pathways after treatment with apatinib.

**Results:**

Treatment with apatinib could induce loss of cell viability of glioma cells, but not of normal astrocytes, through eliciting ferroptosis in vitro and in vivo. It was also revealed that apatinib triggered ferroptosis of glioma cells via inhibiting the activation of nuclear factor erythroid 2-related factor 2/vascular endothelial growth factor receptor 2 (Nrf2/VEFGR2) pathway. The overexpression of Nrf2 rescued the therapeutic effects of apatinib.

**Conclusion:**

Our study proved that treatment with apatinib could restrain proliferation of glioma cells through induction of ferroptosis via inhibiting the activation of VEGFR2/Nrf2/Keap1 pathway. Overexpression of Nrf2 could counteract the induction of ferroptosis by apatinib.

## 1. Background

Glioma is a globally well-known primary malignant tumor characterized by a poor prognosis and a high rate of mortality [1, 2]. Induction of tumor cell death is the main choice for cancer therapy. Ferroptosis is a Fe-dependent regulatory cell death that results from fatal lipid peroxidation [3]. Recently, a number of scholars pointed out that the expression levels of Fe metabolism-related proteins and the activities of Fe-related enzymes were elevated in glioma tissues compared with those in normal tissues [4]. However, very little is currently known about the inducing factors and corresponding outcomes of ferroptosis in glioma.

Apatinib is a vascular endothelial growth factor receptor 2 (VEGFR-2) inhibitor that is successfully used for the treatment of diverse types of cancer [5, 6]. Apatinib possesses antiangiogenic and anticancer activities with different mechanisms. For instance, apatinib treatment could inhibit cell growth and metastasis and enhance the antitumor activity of temozolomide in glioma [7]. However, the efficacy of apatinib has not been fully documented yet. It has been confirmed that apatinib could inhibit proliferation of gastric cancer cells by inducing glutathione peroxidase 4- (GPX4-) mediated ferroptosis [8]. However, the potential mechanisms of apatinib in the treatment of glioma remain elusive.

In the present study, we explored the mechanism of inhibition of glioma cell proliferation by apatinib. It was found that apatinib could arrest cell cycle at G0/G1 phase and inhibit the proliferation of glioma cells through inducing ferroptosis in vitro and in vivo. Of note, nuclear factor erythroid 2-related factor 2 (Nrf2) pathway was closely associated with ferroptosis. A number of scholars have confirmed that Nrf2-associated antioxidant stress plays a key role in ferroptosis inhibition [9]. Indeed, we showed that apatinib inhibits the activation of Nrf2 pathway allowing the induction of ferroptosis. Accordingly, the overexpression of Nrf2 could reverse the inhibition of proliferation and the induction of ferroptosis of glioma cells by apatinib. Therefore, our study revealed a new ferroptosis-related mechanism of apatinib inhibiting proliferation of glioma cells, which facilitated further study on inhibitory effects of apatinib on cancer cells.

## 2. Materials and Methods

### 2.1. Cell Lines and Transfection with Drugs or Plasmids

Human glioma U251 and U87 cells were obtained from Procell Life Science & Technology Co., Ltd. (Wuhan, China). Cells were cultured in a Dulbecco's modified Eagle's medium (DMEM; Gibco, Grand Island, NY, USA) supplemented with 10% fetal bovine serum (FBS) (Gibco) and 1% penicillin-streptomycin solution (Gibco). Glioma cells were transfected with apatinib after reaching 50% confluence according to the manufacturer's protocol. For plasmid transfection, cells were seeded and transfected with the plasmids when they reached 70% confluence using Lipofectamine 2000 reagent (Invitrogen, Carlsbad, CA, USA), according to the manufacturer's protocol.

### 2.2. Cell Counting Kit-8 (CCK-8) Assay

CCK-8 assay was performed to evaluate cell viability. Briefly, U251/U87 cells in the logarithmic growth phase were seeded into a 96-well plate at a density of 5 × 103 cells/well. At the same time, control group was set, and the controls were cultured overnight at 37°C (100 *μ*L sterile phosphate-buffered saline (PBS) was added into the well). Cells were then treated with apatinib for 24, 48, and 72 h, respectively. After that, 10 *μ*L CCK-8 solution (MCE, USA) was added to each well and cultured at 37°C for 4 h; the absorbance value of each well was determined at a wavelength of 450 nm by a miroplate reader.

### 2.3. Cell Cycle Assay

The density of U87 and U251 cells in the logarithmic growth phase was adjusted to 1 × 105 cells/mL, and then the cells were seeded into a 6-well plate. After the cells adhered to the wall, the original medium was discarded, and the cells were treated. The cells were then incubated in an incubator at 37°C for 72 h in the presence of 5% CO2. Subsequently, the supernatant was removed, and the cells were washed with PBS twice. 700 *μ*L precooled 80% ethanol was slowly added until a final concentration of 70%, and the cells were fixed at 4°C for more than 4 h. Subsequently, the cells were centrifuged at 1,500 rpm for 5 min, and RNase (1 mg/mL) was incubated at 37°C for 30 min. 10 *μ*L propidium iodide (PI, 400 *μ*g/mL) solution was added and stained in dark at 4°C for 30 min, and flow cytometry was performed.

### 2.4. Detection of Reactive Oxygen Species (ROS), Malondialdehyde (MDA), Glutathione (GSH), Lactate Dehydrogenase (LDH), and Fe

The density of U87 and U251 cells in the logarithmic growth phase was adjusted to 1 × 105 cells/mL, and then, the cells were seeded into a 6-well plate with 2 mL cell suspension in each well. For ROS detection, the treated cells were analyzed by flow cytometry using a DCFH-DA cell ROS detection kit (Cat. No. S0033; Beyotime Biotechnology, Shanghai, China), according to the manufacturer's instructions. For MDA detection, the treated cells were analyzed by an MDA detection kit (Cat. No. S0131S; Beyotime Biotechnology), according to the manufacturer's protocol. For GSH detection, the treated cells were analyzed by a GSH detection kit (Cat. No. A006-1; Nanjing Jiancheng Bioengineering Institute, Nanjing, China), according to the manufacturer's instructions. For LDH detection, the treated cells were analyzed by a LDH detection kit (Cat. No. A020-1; Nanjing Jiancheng Bioengineering Institute), according to the manufacturer's instructions. For iron detection, the treated cells were analyzed by an iron detection kit (Cat. No. A039-1; Nanjing Jiancheng Bioengineering Institute) and the pcDNA3.1 vector by used (Cat. No.V38520; Invitrogen™).

### 2.5. Western Blotting

Whole-cell protein extracts were homogenized in lysis buffer and centrifuged at 12,000 × g for 15 min. Protein concentration was measured using a BCA Protein Assay Kit. The protein lysates were separated on a 10% sodium dodecyl sulfate- (SDS-) polyacrylamide gel and then transferred onto polyvinylidene fluoride (PVDF) membranes (Millipore, Bedford, MA, USA). After blocking with 5% bovine serum albumin (BSA) for 1.5 h at room temperature, the PVDF membranes were incubated with primary antibodies overnight at 4°C. After washing, the PVDF membranes were incubated with the corresponding secondary antibodies conjugated to horseradish peroxidase. Signals were detected using a commercial ECL kit (Thermo Fisher Scientific, Waltham, MA, USA). Antiglyceraldehyde 3-phosphate dehydrogenase (GAPDH) (Cat. No. 26415-1-AP) was purchased from Proteintech (Chicago, IL, USA). Anti-GPX4 (Cat. No. DF6701), anti-SLC7A11 (Cat. No. DF12509), and inti-phospho-VEGFR2 (Cat. No. AF3279) were purchased from Affinity Biosciences (Cincinnati, OH, USA). Anti-KEAP1 (Cat. No. GTX60660) was purchased from GeneTex Inc. (Irvine, CA, USA). Anti-NRF2 (Cat. No. Ab137550) was purchased from Abcam (Cambridge, UK).

### 2.6. Hematoxylin-Eosin (HE) Staining and Immunohistochemistry

The gliomas from the nude mice were fixed in 10% paraformaldehyde at 4°C for 12 h and then dehydrated in different concentrations of ethanol. The tumor tissues were permeabilized using xylene and embedded in paraffin. They were then sliced (0.5 *μ*m), rehydrated, and stained with HE at 4°C for 10 min and sealed. For IHC assessment of Ki-67 in gliomas, the DAKO Envision system (Dako; Agilent Technologies, Inc.) was used. Briefly, the paraffin-embedded sections of gliomas were heated at 60°C and then incubated with primary antibody against Ki-67 (1 : 1,000; cat. no. ab279653; Abcam) overnight at 4°C. The sections were then incubated with biotin-labeled secondary antibodies (1 : 1,000; cat. no. ab205718; Abcam) at 37°C for 20 min. For evaluation of Ki67, the number of positive cells was calculated in three representative areas of high staining under a light microscope.

### 2.7. Animal Studies

Female BALB/c nude mice (age, 4 weeks old) were purchased from Changzhou Cavens Experimental Animal Co., Ltd. (Changzhou, China). The experimental procedures in this study were performed according to our institutional guidelines for animal experiments, and the protocol was approved by the Institutional Animal Care and Use Committee of Zhejiang Cancer Hospital (Hangzhou, China).

### 2.8. Statistical Analysis

Data were presented as the mean ± standard error of the mean (SEM) from three independent assays. The Student's *t*-test was used to analyze differences between various groups using the GraphPad Prism 6.0 software (GraphPad Software Inc., San Diego, CA, USA). A two-tailed *P* < 0.05 was considered statistically significant (^∗^*P* < 0.05, ^∗∗^*P* < 0.01, and ^∗∗∗^*P* < 0.001).

## 3. Results

### 3.1. Apatinib Causes Loss of Cell Viability through Induction of Ferroptosis of Glioma Cells

**Figure 1:**
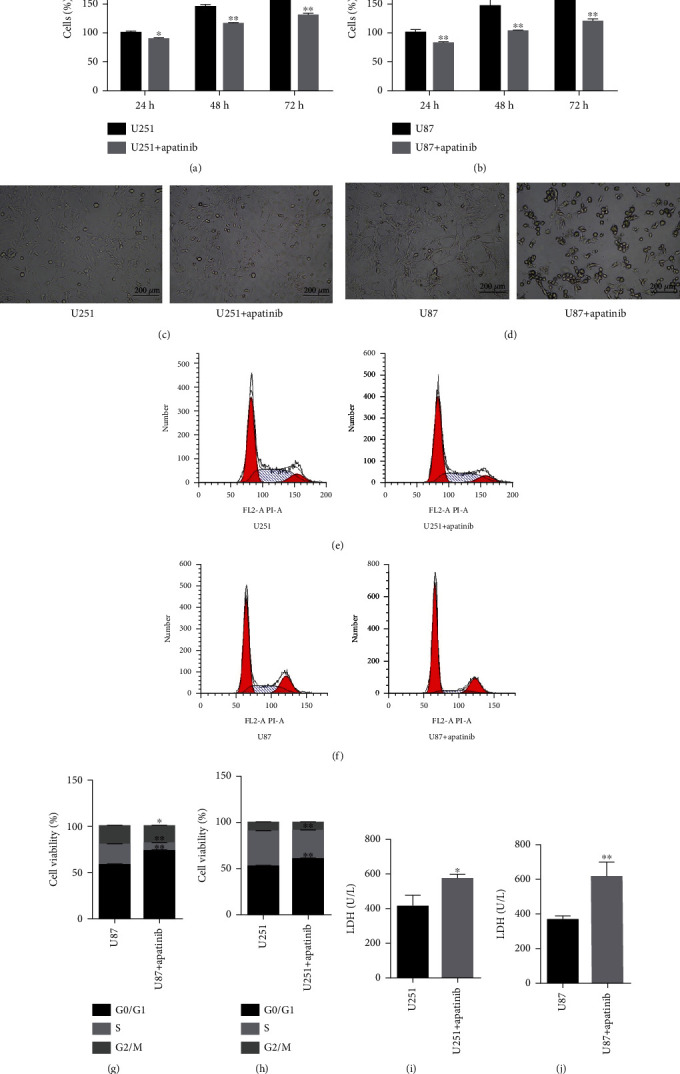
Apatinib induces loss of cell viability and promotes cell cycle arrest at G_0_/G1 phase. (a) CCK-8 assay showed the survival rate of U251 cells treated with apatinib for 24, 48, and 72 h. (b) CCK-8 assay indicated the survival rate of U87 cells treated with apatinib for 24, 48, and 72 h. (c) Representative images of the morphology of U251 cells by an optical microscope. (d) Representative images of the morphology of U87 cells by an optical microscope. (e, f) Representative histograms of cell cycle analysis of U251 and U87 cells 72 h after the absence or presence of apatinib. (h)–(k) Quantitative analysis of cell cycle phases in U251 and U87 cells 72 h after treatment with apatinib. (i, j) LDH levels in the supernatant of U251 and U87 cells without treatment and 72 h after treatment with apatinib 72 h. ^∗^*P* < 0.05; ^∗∗^*P* < 0.01.

Glioma U251 and U87 cell lines were used in the current study. After treatment with apatinib for 24, 48, and 72 h, the survival rate of U251 and U87 cells significantly decreased, and the effect was the most significant at 72 h (Figures 1(a) and 1(b)). Thus, 72-hour treatment with apatinib was selected for further study. The cell morphology was also observed by an optical microscope. After treatment with apatinib for 72 h, the morphology of U251 cells was disordered, the edge became blurred (Figure 1(c)), and round-shaped U87 cells were observed (Figure 1(d)). Flow cytometry showed that the % of cells at G_0_/G_1_ phase increased, at S phase decreased, and at G_2_/M phase slightly decreased or remained unchanged (Figures 1(e)–1(h)), indicating that the cell cycle progression of U251 and U87 cells was arrested at G_0_/G_1_ phase. After treatment with apatinib for 72 h, the LDH level in the supernatant of U251 and U87 cells significantly increased, indicating cell damage (Figures 1(i) and (j)).

**Figure 2:**
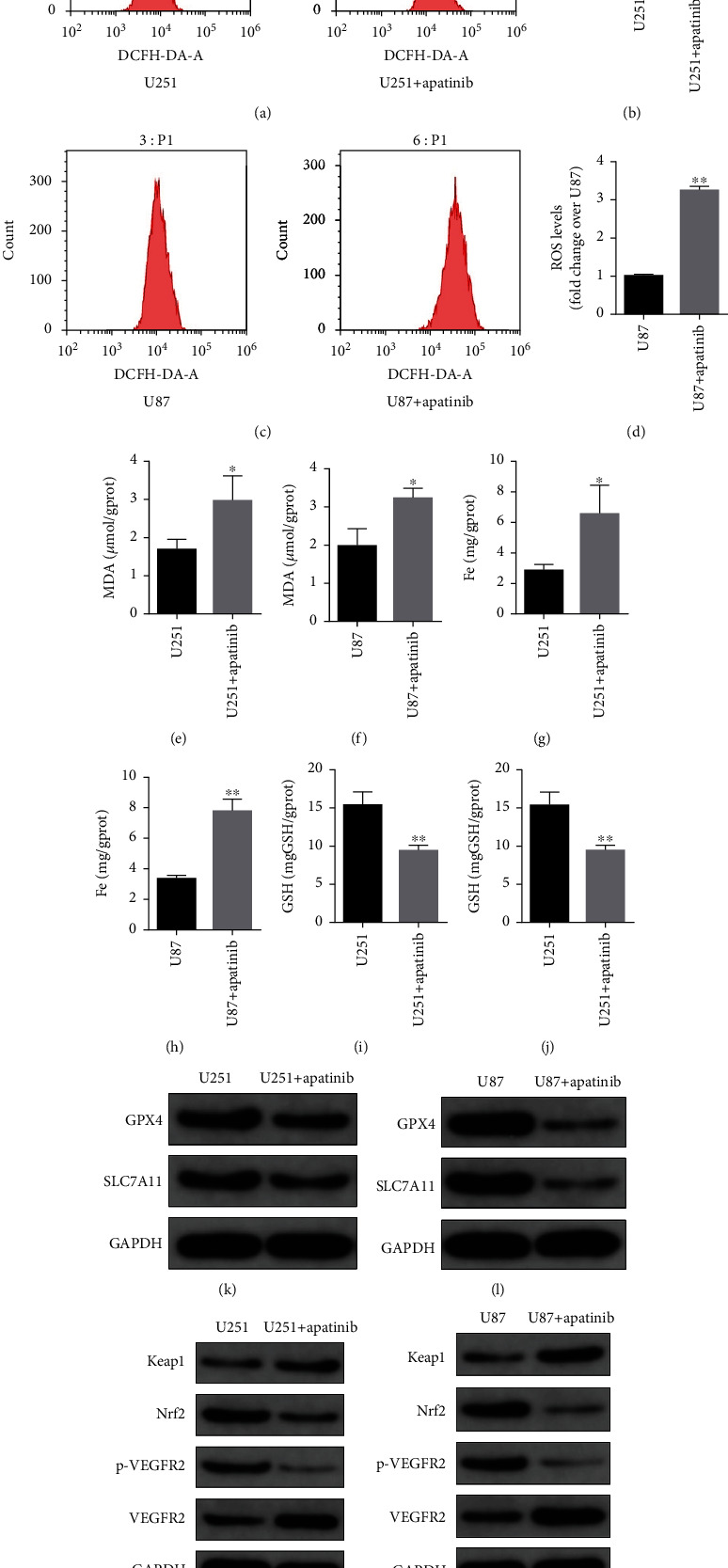
Apatinib induces ferroptosis of glioma cells. All the data refer to 72 h in the presence and in the absence of apatinib. (a) Flow cytometry representative histograms of DCFH-DA-stained U251 cells. (b) Fold change of the mean fluorescence intensity of DCFH-DA-stained U251 cells. (c) Flow cytometry representative histograms of DCFH-DA-stained U87 cells. (d) Fold change of the mean fluorescence intensity of DCFH-DA-stained U87 cells. (e, f) MDA levels in U251 and U87 cells. (g, h) Fe levels in U251 and U87 cells. (i, j) GSH levels in U251 and U87 cells. (k, l) Western blotting was used to determine the levels of GPX4 and SLC7A11 in U251 and U87 cells. (m, n) Western blotting was employed to detect the expression levels of Keap1, Nrf2, p-VEGFR2, and VEGFR2 in U251 and U87 cells. ^∗^*P* < 0.05; ^∗∗^*P* < 0.01.

In addition, after treatment with apatinib for 72 h, the ROS level in U251 and U87 cells significantly increased (Figures 2(a)–2(d)). Moreover, the levels of MDA (Figures 2(e) and 2(f)) and Fe (Figures 2(g) and 2(h)) increased, and GSH levels (Figures 2(i) and 2(j)) decreased in U251 and U87 cells after treatment with apatinib for 72 h. These results indicated that cells were damaged after apatinib treatment. Considering the increase of Fe levels, we checked for ferroptosis status of cells. It was found that the expression levels of GPX4 and SLC7A11 decreased in both U251 and U87 cells after treatment with apatinib for 72 h (Figures 2(k) and 2(l)), indicating the induction of ferroptosis.

### 3.2. Apatinib Induces Ferroptosis through Modulation of VEGFR2/Nrf2/Keap1 Pathway

**Figure 3:**
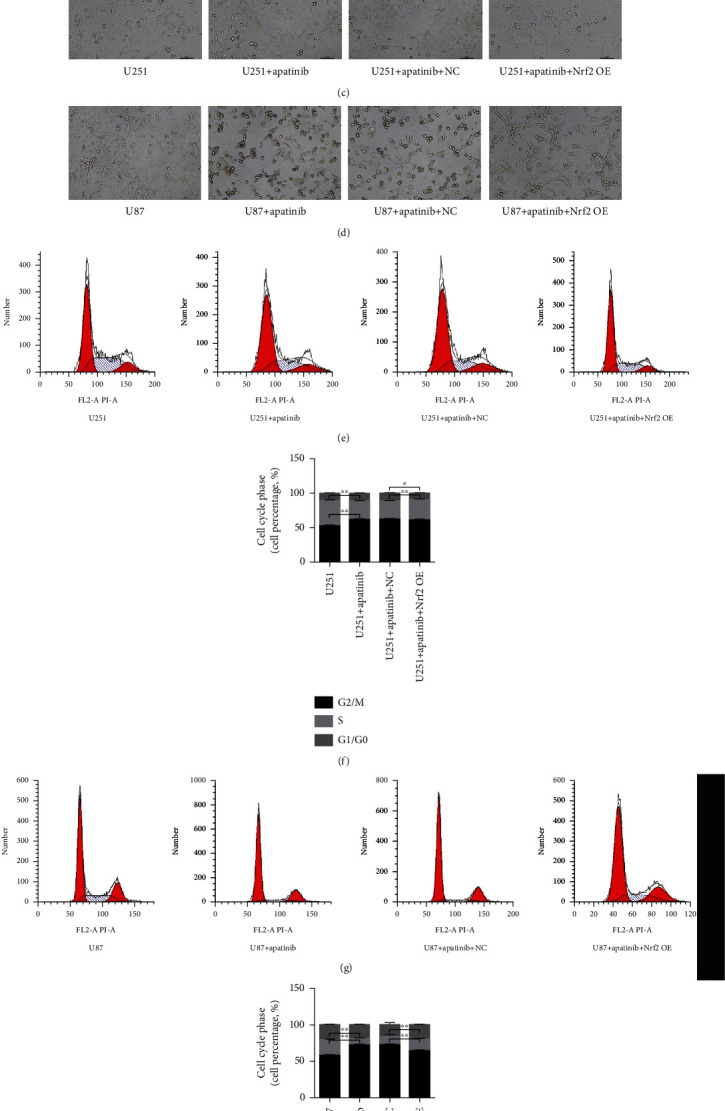
Overexpression of Nrf2 reverts the loss of cell viability and the cell cycle arrest at G_0_/G_1_ phase induced by apatinib. All data refer to of U251 and U87 cells transfected with the empty vector (NC) and overexpressing Nfr2. (a, b) CCK-8 assay shows the cell viability 24, 48, and 72 h after treatment with apatinib. (c, d) Representative images of the morphology of cells by an optical microscopy. (e, g) Representative histograms of cell cycle analysis 72 h after treatment with apatinib. (f, h) Quantitative analysis of cell cycle phases 72-hour after treatment with apatinib. ^∗^*P* < 0.05; ^∗∗^*P* < 0.01.

**Figure 4:**
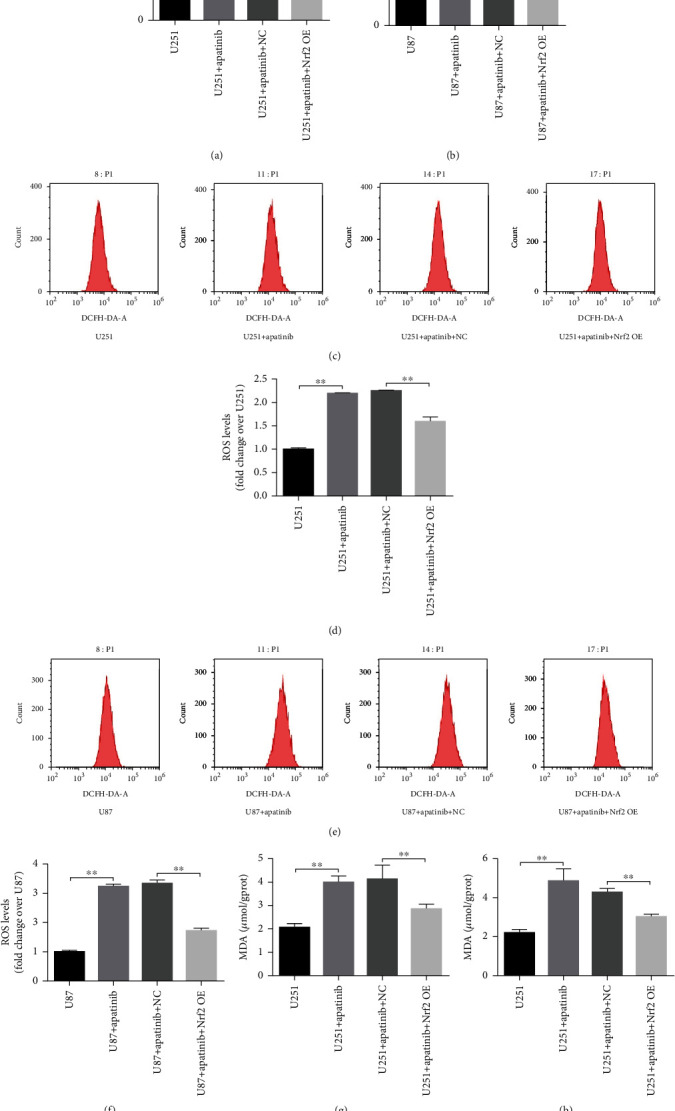
Overexpression of Nrf2 reverts ferroptosis induction by apatinib. All data refer to of U251 and U87 cells transfected with the empty vector (NC) and overexpressing Nfr2 72 h after treatment in the presence or absence of apatinib. (a, b) LDH levels in the cell's supernatant. (c) flow cytometry representative histograms of DCFH-DA-stained U251 cells. (d) Fold change of the mean fluorescence intensity of DCFH-DA-stained U251 cells. (e) Flow cytometry representative histograms of DCFH-DA-stained U87 cells. (f) Fold change of the mean fluorescence intensity of DCFH-DA-stained U87 cells. (g, h) MDA cellular levels. ^∗^*P* < 0.05; ^∗∗^*P* < 0.01.

**Figure 5:**
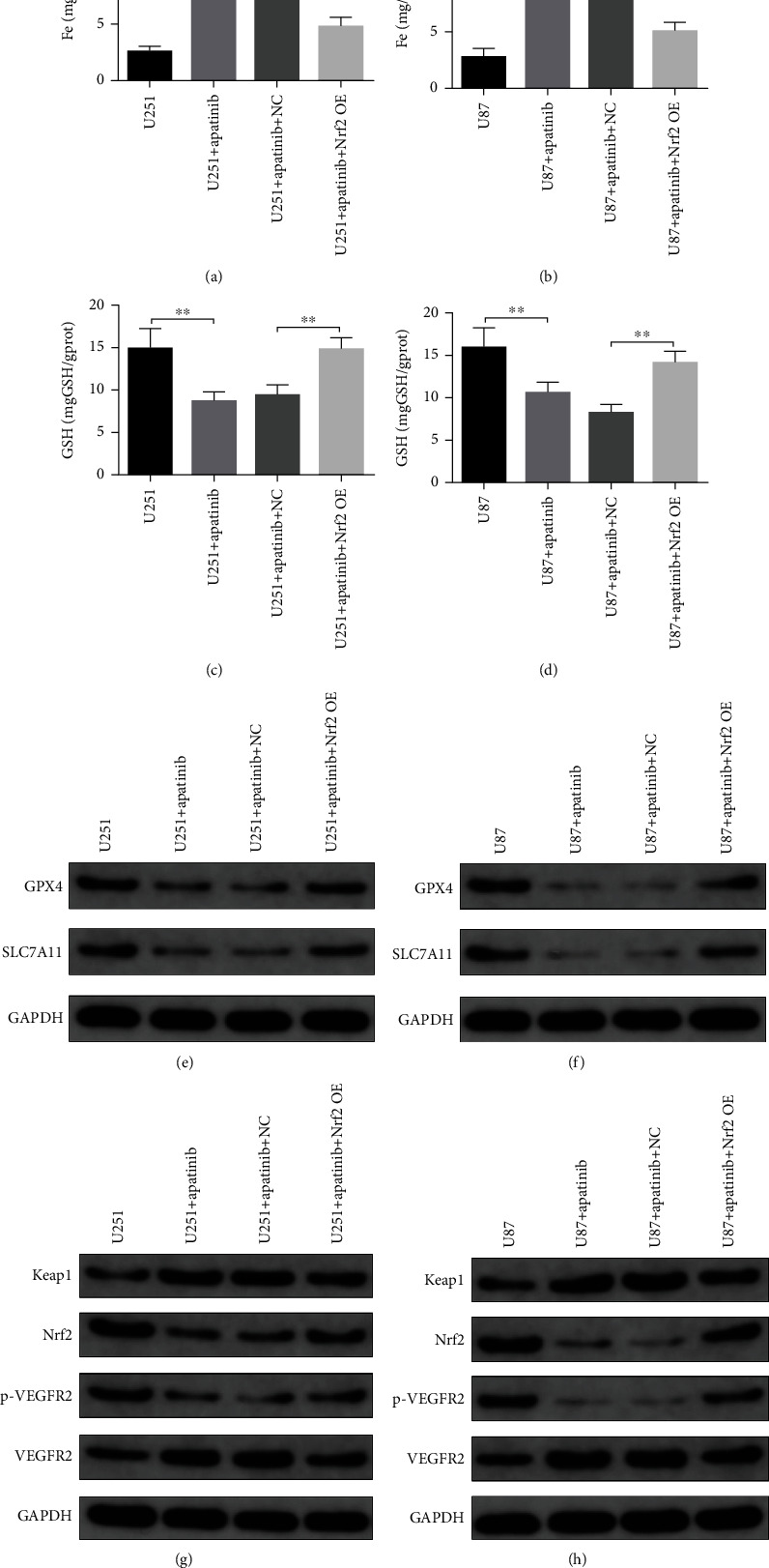
Nrf2 overexpression reverts the increase in iron, the decrease in GSH, GPX4, and SLC/A11, and the changes in expression of the VEGFR2/Nrf2/Keap1 pathway components induced by apatinib. (a, b) Fe levels; (c, d) GSH levels; (e, f) expression levels of GPX4 and SLC7A11; (g, h) expression levels of Keap1, Nrf2, p-VEGFR2, and VEGFR2 in U251 and U87. ^∗^*P* < 0.05; ^∗∗^*P* < 0.01.

We next examined whether apatinib affected the regulatory pathways involved in ferroptosis induction, particularly the VEGFR2/Nrf2/Keap1 pathway. The results revealed that the expression levels of Keap1 and VEGFR2 increased, while the expression levels of Nrf2 and p-VEGFR2 decreased in U251 and U87 cells 72 h after treatment with apatinib (Figures 2(m) and 2(n)). In order to ascertain whether Nrf2 could be involved the response of U251 and U87 cells to apatinib treatment, further analysis was performed with cells overexpressing Nrf2. The results showed that survival of U251 and U87 cells overexpressing Nrf2 remarkably increased 24, 48, and 72 h after treatment with apatinib (Figures 3(a) and 3(b)) with the most significant increase after 72 h, in comparison with cells transfected with the empty vector. The morphology of U251 cells was disordered, and the edge became blurred in the apatinib treatment group, while in the Nrf2 overexpression group, it tended to be normal and the edge was clear (Figure 3(c)). The same trend was observed in U87 cells (Figure 3(d)). Flow cytometry showed that the cell cycle increased at G_0_/G_1_ phase, decreased at S phase, and did not significantly change at G_2_/M phase after treatment with apatinib for 72 h, which indicated that apatinib could block the cell growth at G_0_/G_1_ phase. After overexpression of Nrf2, there was no significant change at G_0_/G_1_ phase, and the cell cycle increased at S phase in U87 cells, indicating that the inhibition of cell growth was relieved. However, results from U251 cells did not show any relevant differences between Nrf2 overexpressing and nonoverexpressing cells treated with apatinib (Figures 3(e)–3(h)). In addition, the levels of LDH (Figures 4(a) and 4(b)), ROS (Figures 4(c)–4(f)), MDA (Figures 4(g) and 4(h)), and Fe (Figures 5(a) and 5(b)) were significantly elevated, while GSH levels (Figures 5(c) and 5(d)) decreased in U251 and U87 cells treated with apatinib for 72 h, indicating that the cells were damaged. On the basis of apatinib treatment, the overexpression of Nrf2 could reduce the effects of apatinib treatment, thereby reducing cell damage. Moreover, the overexpression of Nrf2 in apatinib-treated cells increased the expression levels of GPX4 and SLC7A11 (Figures 5(e) and 5(f)). Additionally, the decrease in Nrf2 and phosphorylated VEGFR2 and the increase in Keap1 in Apatinib-challenged glioma cells were abolished by the Nrf2 overexpression (Figures 5(g) and 5(h)). These results indicated that apatinib could inhibit the proliferation of glioma cells and promote ferroptosis through modulation of VEGFR2/Nrf2 pathway.

### 3.3. *In Vivo* Experiments Confirm That Apatinib Could Promote the Ferroptosis of Glioma Cells

**Figure 6:**
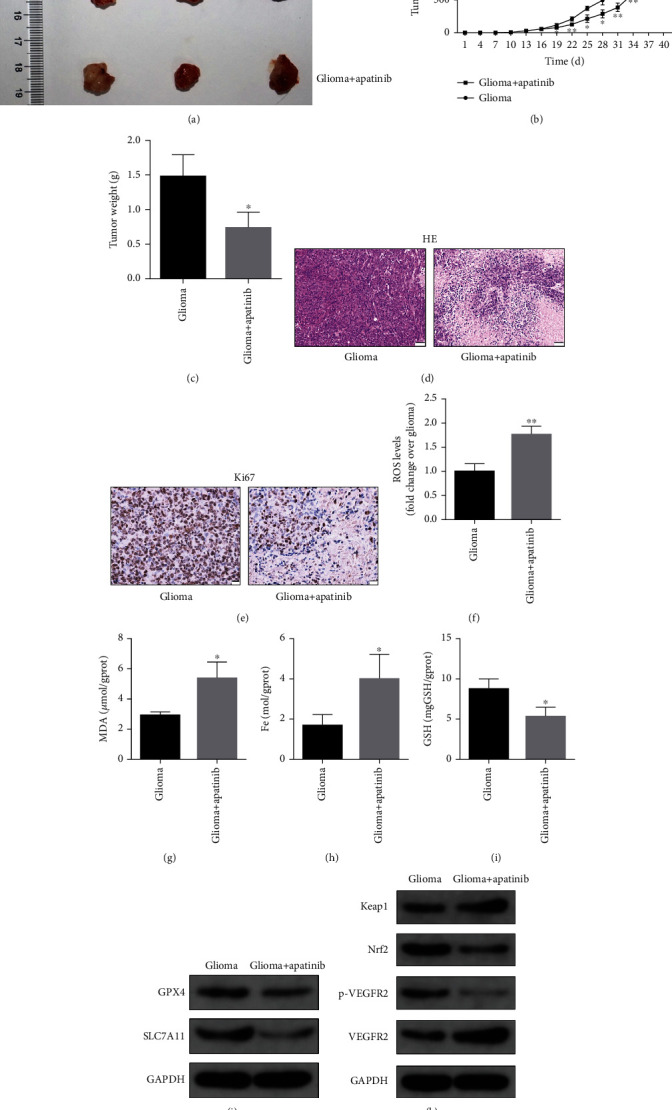
Apatinib promotes *in vivo* ferroptosis of glioma cells. (a) Subcutaneous tumor images of nude mice. (b) The growth curve of subcutaneous tumors. (c) The weights of subcutaneous tumors. (d) Representative hematoxylin-eosin (HE) staining images of subcutaneous tumors. (e) Representative immunohistochemistry images of Ki67 using subcutaneous tumors. Effect of apatinib treatment on subcutaneous tumors, the levels of ROS (f), MDA (g), Fe (h), and GSH (i), expression levels of GPX4 and SLC7A11 (j), and on the expression levels of Keap1, Nrf2, p-VEGFR2, and VEGFR2 (k). ^∗^*P* < 0.05; ^∗∗^*P* < 0.01.

Our results were further verified using a nude mouse xenograft model. The tumor volume of nude mice gradually increased. Compared with the control group, the tumor volume was significantly reduced at each time point after treatment with apatinib (Figures 6(a) and 6(b)). The tumor weight was lighter in the apatinib-treated group compared with that in the control group (Figure 6(c)). Compared with the control group, the proliferation of tumor cells was significantly reduced after treatment with apatinib. Nuclear pyknosis and fibrous tumor tissues appeared in the apatinib-treated group (Figure 6(d)). The percentage of Ki67-positive tumor cells significantly decreased, indicating that the cell proliferation was inhibited (Figure 6(e)). In addition, the levels of ROS, MDA, and Fe in the tumor tissues were significantly elevated, while the GSH level was markedly reduced after treatment with apatinib, indicating that the tumor cells were damaged (Figures 6(f)–6(i)). Western blotting of tumor tissues showed that the expression levels of GPX4 and SLC7A11 were downregulated after treatment with apatinib (Figure 6(j)). The expression levels of Keap1 and VEGFR2 increased, while the expression levels of Nrf2 and p-VEGFR2 decreased in the apatinib-treated group (Figure 6(k)). The abovementioned results indicated that apatinib could promote ferroptosis of glioma cells *in vivo*.

## 4. Discussion

Glioma is a common tumor that originated from the brain, and molecular targeted therapy is one of the important treatment modalities of glioma [10]. Apatinib is a small-molecule tyrosine kinase inhibitor, which is widely used for the treatment of gliomas [7, 11, 12]. For instance, apatinib could inhibit the growth of gastric cancer cells by inducting apoptosis and autophagy [13]. However, the underlying molecular mechanism has still remained mysterious. Induction of tumor cell death is the main choice of cancer treatment. Recently, ferroptosis has been proved as a new type of regulated cell death that could be caused by iron-dependent lipid peroxidation [14]. Triggering ferroptosis of tumor cells has been confirmed as an effective anticancer approach [15].

In the present study, we attempted to investigate the role of ferroptosis in apatinib-involved anticancer mechanism. We first proved that apatinib could inhibit the growth of glioma cells. It has been demonstrated that a high ROS level increases intracellular Fe level and ferroptosis [16]. For instance, RSL3 could drive ferroptosis by inactivating GPX4 and producing ROS [17]. The levels of ROS and Fe were measured, as well as of cellular changes typical of ferroptosis indicating that the treatment with apatinib committed glioma cells to this type of regulated death.

A number of scholars pointed out that Keap1/Nrf2 signaling pathway could regulate the activation of ferroptosis [18]. HMGB1, for instance, could regulate ferroptosis through activation of Keap1/Nrf2 signaling pathway in mesangial cells [19]. In addition, Nrf2 overexpression or Keap1 knockdown could accelerate the proliferation and oncogenic transformation of glioma cells [20]. Nrf2-Keap1 pathway was also proved to diminish ferroptosis [21, 22]. To explore the potential mechanism of apatinib regulating ferroptosis, we determined the expression level of Nrf2 in glioma cells treated with apatinib. The *in vitro* and *in vivo* results revealed that the expression levels of Nrf2 and p-VEGFR2 decreased in cells and tumor tissues treated with apatinib. Moreover, the overexpression of Nrf2 could reverse the induction of ferroptosis and inhibition of cell proliferation by apatinib in the apatinib-treated group. These results indicated that apatinib could promote ferroptosis of glioma cells via modulation of the Keap1/Nrf2 signaling pathway.

## 5. Conclusions

In summary, our study indicated that apatinib could inhibit proliferation of glioma cells by induction of ferroptosis. In terms of the underlying mechanism, it was proved that Keap1/Nrf2 signaling pathway mediated this process. Therefore, the results of the present research revealed a new mechanism of apatinib inhibiting proliferation of glioma cells, which facilitated further study on the inhibitory effects of apatinib on cancer cells.

## Data Availability

The authors confirm that the data supporting the findings of this study are available within the article.

## Abbreviations

VEGFR-2: Vascular endothelial growth factor receptor 2
GPX4: Glutathione peroxidase 4
DMEM: Dulbecco's modified Eagle's medium
FBS: Fetal bovine serum
HMGB1: High-mobility group box 1 protein.

## Supplementary Materials

Supplementary Materials Graphical abstract Apatinib induced ferroptosis in glioma cells via inhibiting the activation of Nrf2/VEFGR2 pathways and thereby induced loss of cell viability of glioma cells.
