# Supplementary figures and images for: Apatinib Induces Ferroptosis of Glioma Cells through Modulation of the VEGFR2/Nrf2 Pathway
Source: Oxid Med Cell Longev. 2022 May 11;2022:9925919. doi: 10.1155/2022/9925919 (PMC9117021; doi:10.1155/2022/9925919)

Graphical abstract

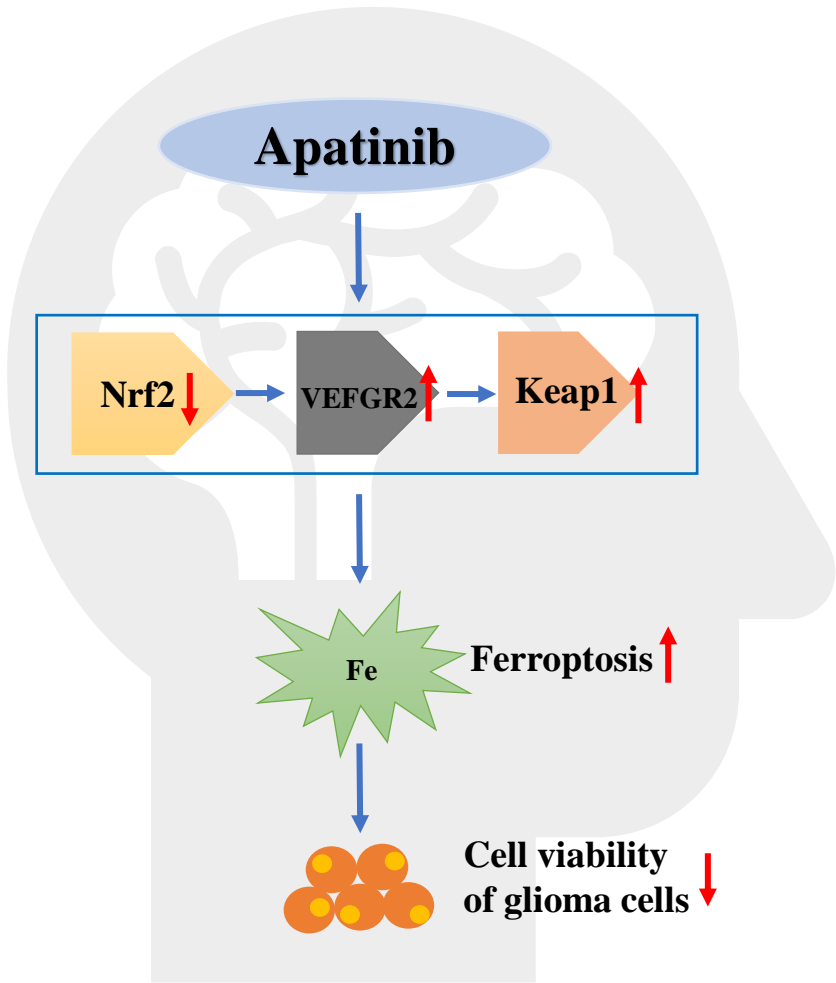

Supplement: Supplementary Materials — Graphical abstract Apatinib induced ferroptosis in glioma cells via inhibiting the activation of Nrf2/VEFGR2 pathways and thereby induced loss of cell viability of glioma cells. [file 9925919.f1.pdf]
